# Insights into the Neuroprotective Potential of Epicatechin: Effects against Aβ-Induced Toxicity in *Caenorhabditis elegans*

**DOI:** 10.3390/antiox13010079

**Published:** 2024-01-08

**Authors:** Begoña Ayuda-Durán, Lidia Garzón-García, Susana González-Manzano, Celestino Santos-Buelga, Ana M. González-Paramás

**Affiliations:** Grupo de Investigación en Polifenoles (GIP-USAL), Campus Miguel de Unamuno, Universidad de Salamanca, 37007 Salamanca, Spain; bego_ayuda@usal.es (B.A.-D.); lidiagarzon@usal.es (L.G.-G.); susanagm@usal.es (S.G.-M.)

**Keywords:** neuroprotection, flavonoids, chemotaxis, gene expression, paralysis

## Abstract

Medical therapies to avoid the progression of Alzheimer’s disease (AD) are limited to date. Certain diets have been associated with a lower incidence of neurodegenerative diseases. In particular, the regular intake of foods rich in polyphenols, such as epicatechin (EC), could help prevent or mitigate AD progression. This work aims to explore the neuroprotective effects of EC using different transgenic strains of *Caenorhabditis elegans*, which express human Aβ_1-42_ peptides and contribute to elucidating the mechanisms involved in the effects of EC in AD. The performed assays indicate that this flavan-3-ol was able to reduce the signs of β-amyloid accumulation in *C. elegans*, improving motility and chemotaxis and increasing survival in transgenic strain peptide producers compared to nematodes not treated with EC. The neuroprotective effects exhibited by EC in *C. elegans* could be explained by the modulation of inflammation and stress-associated genes, as well as autophagy, microgliosis, and heat shock signaling pathways, involving the regulation of *cpr-5*, *epg-8*, *ced-7*, *ZC239.12*, and *hsp-16* genes. Overall, the results obtained in this study support the protective effects of epicatechin against Aβ-induced toxicity.

## 1. Introduction

Alzheimer’s disease (AD) is a progressive neurodegenerative disorder responsible for most cases of dementia that is characterized by mental memory loss and cognitive impairment [1]. Although its etiology is unknown, the development in the brain of extracellular β-amyloid (Aβ) plaques and intracellular protein Tau aggregation forming neurofibrillary tangles are hallmarks of this neuropathology [2]. The prevalence of AD has increased concurrently with the extension of life expectancy [1]. However, medical therapeutics to avoid the progression of this disease are limited to date. In this context, the search for alternatives is crucial to prevent and restrain AD. Several studies suggest that certain diets could be related to a reduced risk of suffering from AD. Among them is the Mediterranean diet, which is rich in plant phenolic compounds and has been associated with a lower risk of major chronic degenerative diseases and slower cognitive decline [3].

Plant phenolic compounds, also referred to as polyphenols, are a large group of plant secondary metabolites with a diversity of chemical structures commonly classified into two major classes: flavonoids and non-flavonoids [4]. Epicatechin, belonging to a group of flavan-3-ols, is one of the main flavonoids in the diet, being present in many fruits and vegetables and derived foods such as green tea, chocolate, and red wine [5].

Different mechanisms have been proposed to explain the neuroprotective effects associated with the intake of polyphenols, standing out for their antioxidant activity [6]. However, it is currently accepted that the effects of flavonoids could be related to the modulation of several cellular pathways, as well as the capacity to change gene expression and the redox environment, receptor functions, or enzyme activities [3,7,8]. Specifically, in neurodegenerative disorders, it has been reported that polyphenols could modulate the processing of amyloid precursor protein by activating α-secretase and inhibiting β- and γ-secretase, which would prevent Aβ aggregation and promote disruption of preformed fibrils [9]. Furthermore, they could limit the accumulation of some peptides involved in neurodegenerative disorders, enhancing the clearance of misfolded proteins through the proteostasis network activity [10] and contributing to combat autophagy deficits [11]. The impact of polyphenols on different signaling cascades in response to stress, such as phosphoinositide 3-kinase (PI3K)/protein kinase B (Akt), mitogen-activated protein kinase (MAPK), nuclear factor erythroid 2-related factor 2 (Nrf2), and nuclear factor kappa B (NFkB), has been reported [12]. The modulation of these pathways is important due to their role in neuronal survival, apoptosis, neuronal growth factor-induced mitogenesis, differentiation, and neuronal plasticity [13]. A reduction in gliosis and neuroinflammatory markers has also been described in different in vivo models of AD treated with flavonoids [14]. Furthermore, polyphenols might attenuate mitochondrial dysfunction and hyperphosphorylation of Tau proteins [15,16].

*Caenorhabditis elegans* is a free-living soil worm that presents a high degree of homology and conservation with human metabolic and disease pathways, which offers promising possibilities for studying Alzheimer’s disease [17] and the mechanisms subjacent to the biological activity of natural compounds [18]. There are several strains of *C. elegans* designed to express human pathologic proteins (i.e., abnormal Aβ and Tau aggregates) [17], which have been employed to evaluate the anti-Alzheimer effects of plant extracts or pure polyphenols [19,20,21].

In the present study, the neuroprotective effects of epicatechin (EC) were explored using transgenic strains of *Caenorhabditis elegans*, namely CL4176 and CL2006, which express the human Aβ_1-42_ gene in body wall muscle cells [22,23], and CL2355 that expresses Aβ in neurons exhibiting defects in chemotactic behavior [24]. Additionally, the influence of EC in autophagy, inflammation, and oxidative stress, modulating signaling pathways and the capacity to modulate proteasomal activity, was assessed. These studies aim to gain further insights into the mechanisms involved in the effects of EC in AD. The observations made in the *C. elegans* models of Alzheimer’s disease revealed the existence of beneficial effects of epicatechin against Aβ-induced toxicity, which could be explained by its ability to act on intracellular routes and targets, such as autophagy, microgliosis, inflammation, stress-related factors, or heat shock signaling, specifically modulating the expression of genes like *cpr-5*, *epg-8*, *ced-7*, ZC239.12, and *hsp-16.2*.

## 2. Materials and Methods

### 2.1. Standards and Reagents

(−)-Epicatechin (≥90% HPLC) and benzaldehyde (≥99%) standards, ampicillin sodium salt, nystatin, agar, yeast extract, phosphate-buffered saline (PBS), fluorodeoxyuridine (FUdR), sodium azide, peptone ethanol, and cholesterol were purchased from Sigma-Aldrich (Madrid, Spain). Sodium chloride, calcium chloride, 10% *w*/*v* sodium hypochlorite solution, and dimethyl sulfoxide (DMSO) were obtained from Panreac (Barcelona, Spain). Potassium dihydrogen phosphate, potassium monohydrogen phosphate, sodium monohydrogen phosphate, and magnesium sulphate were obtained from Merck (Darmstadt, Germany). Petri plates Ø 35 and 60 mm were purchased from Brand GMBH (Wertheim, Germany). MRS broth and NP-40 were sourced from Fisher Scientific (Madrid, Spain), and tryptone medium was obtained from Fluka Analytical (Madrid, Spain).

### 2.2. Strains

The mutant strains CL4176, dvIs27[pAF29(myo-3/A-Beta 1-42/let UTR) + pRF4(rol- 6(su1006))]; CL2006, dvIs2 [pCL12(unc-54/human Aβ peptide 1-42 minigene) + rol-6(su1006)]; CL2355, dvIs50 [pCL45 (snb-1::Abeta 1-42::3′ UTR(long) + mtl-2::GFP] I; CL2122, dvIs15 [(pPD30.38) unc-54(vector) + (pCL26) mtl-2::GFP], as well as the *E. coli* OP50 strain, were obtained from the Caenorhabditis Genetics Center at the University Minnesota (Minneapolis, MN, USA).

### 2.3. C. elegans Maintenance

Synchronization of worm cultures was achieved by allowing gravid hermaphrodites to lay eggs for 2–3 h on fresh plates. Alternatively, the process can be carried out by treating gravid hermaphrodites with bleach:5 N NaOH (2:1). Eggs are resistant, whereas worms are dissolved in the bleach solution. The suspension was shaken with a vortex mixer for 1 min and kept for a further minute on rest. This process was repeated five times. The suspension was centrifuged (2 min, 9500× *g*). The pellet containing the eggs was washed six times with an equal volume of buffer M9 (3 g KH_2_PO_4_, 6 g Na_2_HPO_4_, 5 g NaCl, 1 mL 1 M MgSO_4_, and H_2_O to 1 L). In both cases, after synchronization, the eggs were incubated on NGM agar plates with or without EC. Epicatechin solution (50 mM, 100 mM, 150 mM, and 200 mM) in DMSO was added to the nematode growth medium during its preparation to obtain 50 µM, 100 µM, 150 µM, or 200 µM final concentration on the plates, respectively. Control plates were also prepared without the flavonoid but containing the same volume of DMSO (0.1% DMSO, *v*/*v*).

CL4176, CL2355, and CL2122 strains were routinely propagated at 16 °C, while wild-type worms and CL2006 strain were propagated at 20 °C on nematode growth medium (NGM) plates, with *E. coli* OP50 as a food source.

### 2.4. Life Span Assay

Age-synchronized CL2006 young larvae were transferred to NGM agar plates with EC 150 µM or control plates with the same percentage of DMSO (0.1%). Worms were grown at 20 °C. Once they reached adulthood, twenty animals per plate were transferred to fresh plates with or without EC 150 µM and containing 150 µM of 5-fluorouracil-2′-deoxyribose (FUdR) to prevent the development of progeny and to avoid overlapping generations. This moment was considered the first time point for the counting of surviving worms [25]. Assays for each condition were performed with at least 100 nematodes (20 worms per plate/5 plates). Surviving worms were transferred onto fresh plates every two days until all of them were scored dead. Dead worms were recognized when they did not respond after gentle touches with a platinum wire. Three independent assays were performed for all conditions.

### 2.5. Chemotaxis Assays

Chemotaxis assays were performed as described by Dosanjh et al. [26]. Synchronized transgenic *C. elegans* CL2355 and its control strain CL2122 were cultured from eggs with or without EC. They were grown at 16 °C for 48 h on solid nematode growth medium (NGM) plates seeded with *E. coli* OP50. Afterwards, the plates were kept at 25 °C for a further 36 h to induce expression of Aβ in worm neurons. Worms were then collected and washed with M9 buffer twice, and 50-70 individuals were transferred to the center of 35 Ø mm NGM plates without food. Spots of 1 µL of 1 M sodium azide + 4 µL of 0.5% benzaldehyde in ethanol were added on two opposite quadrants (“attractant” sites) at the plate side, while spots of 1 μL of sodium azide + 4 μL of 100% ethanol were added on the other two quadrants (control). Plates were incubated at 20 °C for 1 h, and then the number of worms in each quadrant was scored.

The chemotaxis behavior was scored and expressed as the chemotaxis index (CI), determined by subtracting the number of worms in the control area from the number of worms in the attractant area and dividing it by the total number of worms on the plate. Three independent assays were performed in triplicate for all conditions.

### 2.6. Paralysis Assay

The paralysis assay was performed according to the method of Chen et al. [27]. Strain CL4176 maintained at 16 °C was egg-synchronized and cultured in NGM plates containing *E. coli* OP50 and different concentrations of EC (50–200 µM) or without the flavonoid (0.1% DMSO; control). Following incubation at 16 °C for 48 h, the temperature was raised to 25 °C to induce Aβ transgene expression in muscle cells, and then the number of paralyzed worms was scored under the microscope for 24–32 h at 2 h intervals. Worms were considered paralyzed if they did not move or only bent their head after touching. All assays were performed independently three times.

### 2.7. Proteasomal Activity

*C. elegans* proteasomal activity was determined according to Regitz et al. [28] with some modifications, as described below. Synchronized transgenic *C. elegans* CL2006 worms were grown at 20 °C in the presence or absence of EC and fed with *E. coli* for 120 h. Prior to protein extraction, heat stress (35 °C) was applied for 1 h. The worms were collected with M9 buffer, centrifuged at 10,000× *g* for 1 min, and the pellet was suspended in 200 μL of M9. For protein extraction, the pellet was treated with 200 μL lysis buffer NP-40, and to maximize cell breakage, 10 stainless steel beads (2 mm) were added. The mixture was vortex-shaken vigorously and further homogenized seven times in a FastPrep-24 5G (MP Biomedicals, Irvine, CA, United States), with a speed of 5.5 m/s and runtime duration of 10 s five times. The lysates were centrifuged at 13,000× *g* for 15 min at 4 ˚C. Protein concentration in the supernatants was determined using a Nanodrop 2000 spectrophotometer (ThermoFisher Scientific, Waltham, MA, USA). Chymotrypsin-like protease activity in the nematode homogenates was assessed using a Proteasome 20S fluorescent assay Kit (Sigma-Aldrich). The cleavage of the fluorogenic substrate LLVY-R110 by the proteasome generated strong green fluorescence measured at λ_ex_ = 485 nm and λ_em_ = 520 nm over 3 h every 10 min in a microplate reader (FLUOstar Omega, BMG labtceh, Ortenberg, Germany). To determine the specific proteasomal activity, the fluorescence measured in the presence of MG-132 (Z-Leu-Leu-Leu-al) proteasome inhibitor (final concentration 10 µM; Sigma-Aldrich) was subtracted from activities due to overall fluorogenic peptide cleavage. Each fluorescence measurement was corrected in relation to the total protein content of each sample.

### 2.8. RT-qPCR

Synchronized CL4176 eggs were treated with or without 150 μM EC for 120 h and submitted to 25 °C for 32 h. The worms were collected with M9 buffer, centrifuged at 10,000× *g* for 1 min, and the pellet was suspended in 300 μL of M9. Total RNA was extracted using the RNAspin Mini RNA Isolation Kit (GE Healthcare, Buckinghamshire, UK). In order to maximize cell breakage in the first stage of the extraction, 10 stainless steel beads (2 mm) were added. The mixture was vortex-shaken vigorously and further homogenized in a FastPrep-24 5G with a speed of 5.5 m/s and runtime duration of 10 s five times.

Gene expression of *ced-7*, *vha-5*, *cpr-5*, *epg-8*, *hsp-70*, *ZC39.12*, and *hsp-16.2* was analyzed by real-time qPCR in a BioMark HD System (Fluidigm, South San Francisco, CA, USA) using the GE 96.96 Dynamic Array™ integrated fluidic circuit (IFC) and the *act-1* housekeeping gene as an internal control. The primers used are described in Supplementary Table S1 [29,30,31,32]. In the second step, cDNA-specific target preamplification (STA reaction) was performed using the QIAGEN Multiplex PCR kit (Hilden, Germany) according to manufacturer recommendations. Briefly, a 1.25 μL aliquot of each cDNA was mixed with 0.5 μL pooled DeltaGene primers (500 nM), 0.75 μL nuclease-free water, and 2.5 μL Multiplex Mix. Subsequently, each sample was treated with exonuclease I to eliminate unincorporated primers.

Preamplified cDNA was then used for qPCR measurement of each gene using a BioMark HD system following the manufacturer’s instructions. Briefly, a 2.25 μL aliquot of each amplified cDNA was mixed with 2.5 μL of 2X SsoFast EvaGreen Supermix with Low ROX (Bio-Rad, Madrid, Spain) and with 0.25 μL of 20× DNA Binding Dye Sample Loading Reagent (Fluidigm). Individual qRT-PCR primer pairs (100 mM) were diluted 1:10 with Tris-EDTA (2.5 μL total volume) and mixed with 2.5 μL Assay Loading Reagent (Fluidigm). Each sample mix and assay mix was individually pipetted into one sample or assay inlet, respectively, in a Dynamic Array IFC chip (Fluidigm). Subsequent sample/assay-loading was performed with an IFC Controller HX (Fluidigm), and qRT-PCR was performed on the BioMark HD real-time polymerase chain reaction (PCR) reader (Fluidigm) following the manufacturer’s instructions using standard fast cycling conditions and melt-curve analysis, generating an amplification curve for each gene of interest in each sample. The gene expression data were analyzed using the comparative 2^−ΔΔCt^ method with *act-1* as the normalizer [33]. Ten independent experiments were performed.

### 2.9. Statistical Analyses

GraphPad Prism 9.3.0 software (GraphPad Software, Boston, MA, USA) was used for statistical analyses. The Student’s t-test was used, to compare two groups, whereas one-way ANOVA with Tukey’s test was performed to compare multiple groups. For paralysis assays and lifespan, *p*-values were calculated using the log-rank test with the PC software package SPSS (version 23.0; SPSS Inc., Chicago, IL, USA). In every analysis, significant differences were statistically considered at the level of *p* < 0.05.

## 3. Results

### 3.1. EC Has Beneficial Effects on Body Paralysis in the CL4176 Strain

The *C. elegans* transgenic model of Alzheimer’s disease CL4176 was used to determine if EC could protect against Aβ-induced toxicity in vivo. This strain possesses a phenotype that can be easily monitored over time as it presents muscle paralysis when transgene expression is induced by raising the temperature from 16 °C to 25 °C. The score was performed after temperature upshift to 25 °C for 24 h and then every 2 h until 36 h post-upshift.

Preliminary assays were carried out culturing nematodes in NGM media in the presence of different EC concentrations (50–200 μM) and without EC (control) to determine whether protective effects against Aβ proteotoxicity could be observed. Table 1 shows the rates of worm paralysis at the different assayed EC concentrations.

It was observed that a significant delay in worm paralysis was only produced at an EC concentration of 150 µM (log-rank test, *p* < 0.001). Lower EC concentrations (50–100 µM) maintained the same rate of paralyzed worms as non-treated worms, whilst greater EC concentration (200 µM) slightly, but not significantly, increased the delay in Aβ-induced muscle paralysis. While the observed effect did not seem to work in a dose-dependent manner, it might be in agreement with previous studies of our group where a hormetic response in the flavonoid protection against oxidative damage in *C. elegans* was observed, with only a short range of concentrations showing beneficial effects [34,35]. Based on these results, an EC concentration of 150 µM was selected for further assays.

**Figure 1:**
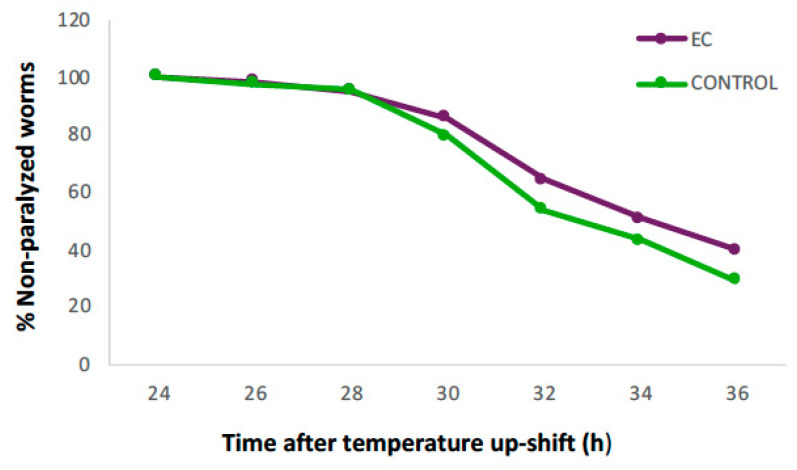
Evolution in the percentage of non-paralyzed worms in the transgenic *C. elegans* CL4176 strain treated with 150 μM of epicatechin (EC) or without EC (control). Following incubation at 16 °C for 48 h, the expression of β-amyloid peptide (Aβ) was induced by raising the temperature to 25 °C. Twenty-four hours after temperature upshift, worms were scored for paralysis at 120 min intervals. Plots were obtained from the mean of three independent experiments (n = 100 worms/group).

Figure 1 shows the evolution in the percentage of non-paralyzed worms treated with 150 µM of EC compared to the control. It can be observed that 24 h after induction of Aβ_1–42_ expression, all animals were still in motion in both groups, but from 28 h onwards, the percentage of non-paralyzed worms in the EC group remained higher until the end of the assay. At 36 h, 40% of EC-treated worms were still non-paralyzed compared to 29% in the control group.

The accumulation of toxic plaques composed mainly of insoluble Aβ is considered the central event triggering neuron degeneration in Alzheimer’s disease. It is also known that oxidative stress plays an important role in the development of neurodegenerative diseases such as Alzheimer’s and Parkinson’s [36]. Natural compounds able to reduce oxidative damage have been related to neuroprotective effects, allowing for the deceleration of the progression of aggregation-related diseases [20,37,38]. In previous studies of our group, it was observed that the treatment of wild-type *C. elegans* with EC led to a significant increase in the survival of the nematode when subjected to oxidative stress, and that also decreased the levels of lipid peroxidation and carbonylated proteins, suggesting protection of EC against oxidative damage [39,40,41]. In the present study, treatment with EC 150 μM induced a significant improvement in body paralysis progression in the Aβ model CL4176 strain, suggesting that this flavonoid can alleviate Aβ toxicity. Some authors explained the anti-AD effects of polyphenols by their ability to prevent the aggregation of Aβ fibrils and accumulation of Tau protein intracellular neurofibrillary tangles [15,42]. It was suggested that the presence of a catechol group in the B ring of flavonoids such as epicatechin or quercetin contributes to inhibiting protein aggregation by reducing the secondary folding of the β-sheet structures characteristic of Aβ plaques and neurofibrillary tangles [42]. Similar to our observations, other studies with the strain CL4176 also reported a significant effect of different phenolic compounds or extracts rich in polyphenols in delaying paralysis. For example, the prenylated flavonols icariin and icariside II were found to delay the onset of paralysis mediated by Aβ_1–42_ proteotoxicity [43]. Likewise, extracts from *Ginkgo biloba* [19], *Baccharis trimera* [37], and Zijuan Pu’er tea [20] were shown to be capable of suppressing Aβ-induced pathological behaviors in transgenic *C. elegans* strains.

### 3.2. Epicatechin Extends the Lifespan of the CL2006 Strain

The transgenic CL2006 strain constitutively expresses human Aβ_1–42_ in the cytoplasm of body wall muscle tissues in an age-dependent way (as it occurs in humans), producing progressive paralysis of the nematode, eventually leading to death [44].

**Figure 2:**
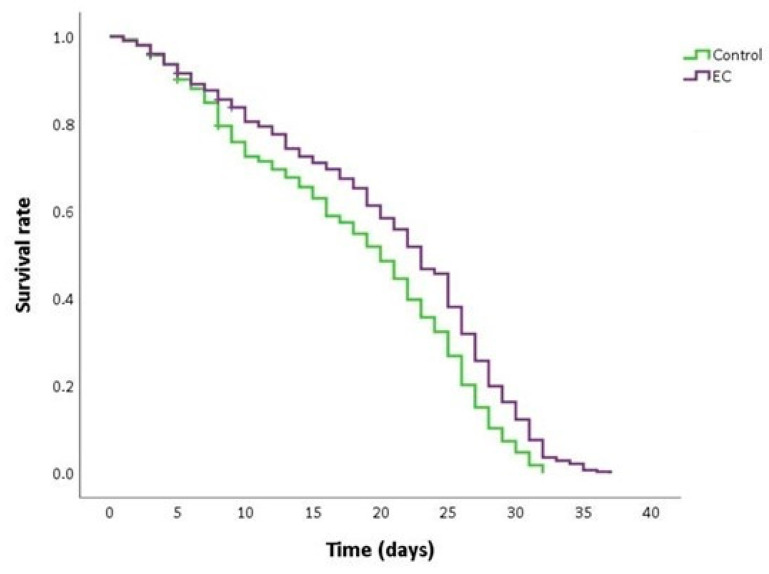
Kaplan–Meier survival curves of CL2006 worms grown at 20 °C in nematode growth medium (NGM) plates supplemented with EC 150 µM or dimethyl sulfoxide (DMSO) (control worms). Plots are representative of three independent experiments (n = 100 worms/experiment).

As can be seen in Figure 2 and Table 2, the treatment with EC 150 µM significantly extended the mean and maximum lifespan and increased the survival rate in CL2006 worms compared to non-treated nematodes. This finding suggests that EC may attenuate Aβ-induced toxicity, thus causing a delay in the progression of paralysis and death. Similar results in the improvement of lifespan in the CL2006 strain by different natural bioactive products were also described by other authors [45,46].

### 3.3. Epicatechin Suppresses Neuronal Aβ Expression-Induced Defects in Chemotaxis Behavior

**Figure 3:**
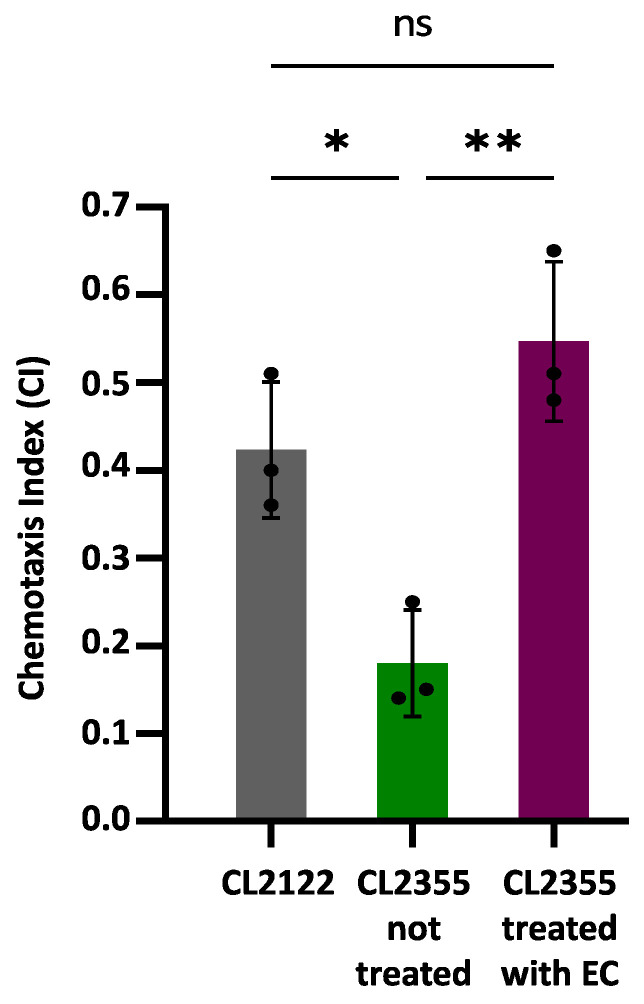
Chemotaxis behavior in neuronal Aβ-expressing strain CL2355 not treated and treated with EC 150 µM and control strain CL2122. In all cases, the results were obtained from three plates containing 50–70 worms each; three individual experiments were carried out in each case. Data are represented as mean ± SD. Dots represent individual data points. Asterisks indicate significant differences: * *p* < 0.05 (CL2122 vs. CL2355 untreated) and ** *p* < 0.01 (CL2355 treated with EC vs. untreated CL2355), ); ns = not significant.

Chemotaxis assays were carried out using the CL2355 transgenic strain that expresses Aβ_1-42_ in neurons, which causes defects in the chemotaxis behavior [26]. The transgenic strain CL2122, presenting a wild-type phenotype under the same assay conditions, was used as a control. Figure 3 shows the obtained results, expressed as the chemotaxis index (CI), which are indicative of the number of worms that successfully move towards the attractant benzaldehyde.

The CI value was significantly reduced in the untreated CL2355 strain (CI = 0.18 ± 0.06; *p* = 0.007) compared to the control strain CL2122 (CI = 0.42 ± 0.08). However, the treatment with 150 µM EC normalized the chemosensory response of CL2355 (CI = 0.55 ± 0.09), significantly increasing the CI compared to untreated CL2355 worms (*p* = 0.026). These observations suggest that treatment with EC protects in vivo neurons against amyloid toxicity. The improvement in Aβ-induced chemotaxis defects has also been observed in CL2355 treated with natural bioactive products rich in polyphenols, such as extracts of leaves from *Ginkgo biloba* [19] or *Glochidion zeylanicum* [47].

Overall, taking into account that EC delays Aβ-induced onset and aged paralysis in strains which express Aβ in muscle (i.e., CL4176 and CL2006) and improved the chemotaxis behavior in worms that express Aβ in neurons (i.e., CL2355), the obtained results suggest that epicatechin is able to provide in vivo protection against amyloid toxicity both in muscular and neuronal cells. The effects on neurons may be particularly relevant since, in human AD, the small oligomeric forms of Aβ-peptide are considered the main toxic species in these cells [38].

### 3.4. Epicatechin Does Not Modify Proteasomal Activity

Most neurodegenerative diseases present a disruption of protein homeostasis [48]. There are protein quality control systems that can repair or degrade the damaged protein, preventing excessive protein misfolding and degradation (proteostasis). When the mechanisms do not respond to proteotoxic imbalances, the misfolded protein could accumulate, leading to neurodegeneration [49].

**Figure 4:**
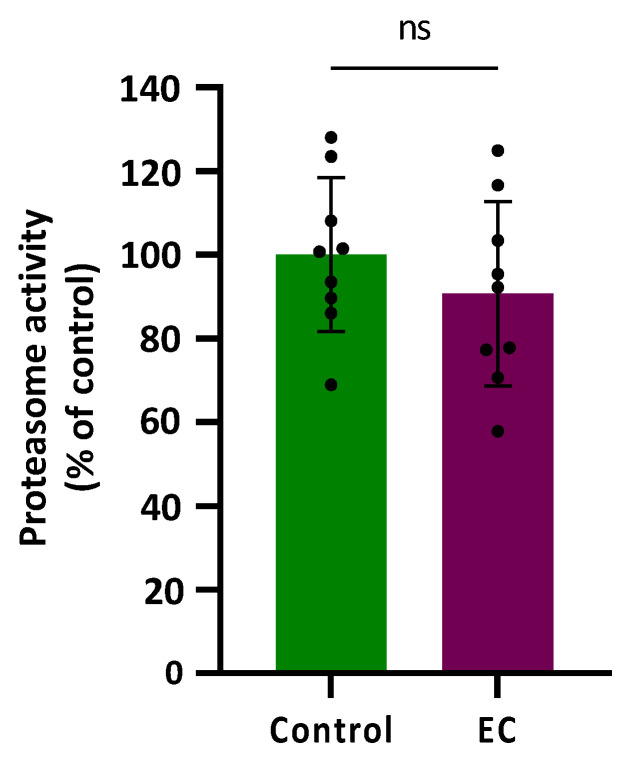
Plots and bars represent the results obtained for the proteasomal activity in transgenic CL2006 worms treated (EC) and not treated (control) with epicatechin 150 µM. The results are expressed as a % of response in relation to the control and presented as mean ± SD (n = 9). Dots represent individual data points; ns = not significant.

Proteasomal activity was assessed in CL2006 worms treated (120 h) or not with EC 150 µM by determining the difference in cleavage of the fluorogenic peptide Suc-LLVY-AMC in the presence or absence of the proteasome-specific inhibitor MG132 at 10 µM. The results are shown in Figure 4 expressed as a % compared with the average value obtained in control worms (not treated) that was assigned as 100%. As can be observed, the proteasome activity was not modified significantly (*p* = 0.343) by treatment with epicatechin 150 µM.

In the previous assays, it was found that EC reduced paralysis and increased survival in CL4176 and CL2006 strains expressing the human Aβ peptide in body wall muscle cells and rescued neuronal Aβ expression-induced defects in chemotaxis behavior (CL2355 strain). Those observations seemed to indicate that EC attenuates the toxicity induced by Aβ. However, in this assay in the strain CL2006, no improvement in the proteasomal activity following EC treatment was observed. This finding might be explained by the existence of other ways to remove non-functional proteins besides proteasomal activity, such as protein refolding in the endoplasmic reticulum (ER), mitochondrial unfolding protein response, chaperone activation, or degradation of proteins through autophagy [49,50]. It is also likely that exposure to EC led to the activation of endogenous mechanisms of defence against oxidative damage so that, ultimately, these worms would not require further induction of protein degradation. In fact, in previous studies of our group, it was shown that treatment with EC reduced oxidative damage of biomolecules and increased survival in wild-type *C. elegans* submitted to thermal stress, which was explained by the overexpression of some stress resistance-associated genes, such as *gst-4*, *hsp-16.2*, and *hsp-70* [40]. Actually, the mechanisms behind the effects of the flavonoids are still under discussion and the possibility that flavonoids could modify gene expression or act as potential modulators of intracellular signaling cascades are now gaining strength [6].

### 3.5. Influence of EC on Gene Expression

#### 3.5.1. Selection of Genes

In order to explore the molecular mechanisms that could be behind the protective effects observed for EC, the expression of several genes involved in cell signaling pathways related to hallmarks of the disease (e.g., amyloid deposits, neurofibrillary tangles, synaptic loss, autophagy deficits, inflammation, and extensive oxidative stress) was analyzed. In particular, the genes *vha-5*, *cpr-5*, *epg-8*, *ced-7*, *ZC239.12*, *hsp-16.2*, and *hsp-70* were selected based on results from our previous studies [40,51] and data obtained from the literature, as below described.

Autophagy is a lysosomal degradative process used to recycle cellular waste and eliminate potentially toxic damaged organelles and protein aggregates. Defective lysosomal acidification (caused by aging) has been identified as a cause of abnormal autophagic flux and contributes to proteolytic failure in several diseases, including Alzheimer’s disease [52]. Thus, in the context of autophagy modulation, the focus was put on genes such as *vha-5*, *cpr-5*, and *epg-8*; *vha-5* is a gene that encodes vacuolar (H^+^)-ATPase, which maintains lysosomal acidity and degradation competence [53], while *cpr-5* is an ortholog of human cathepsin B (CatB), a cysteine-type endopeptidase that is located in lysosomes and promotes cellular proteolysis [54]. Furthermore, the expression of *epg-8*, a gene related to the PI3K complex, was also checked, as it has been described that autophagosome reduction by the phosphoinositide 3-kinase (PI3K) complex delayed Aβ-induced paralysis in the CL4176 strain and alleviated Aβ-induced toxicity, causing a neuroprotective effect [30].

Currently, microgliosis and chronic inflammation are well-accepted as pathological hallmarks of Alzheimer’s disease. During AD progression, microglia (including phagocytosis) could turn into a dysfunctional pro-inflammatory phenotype, which ultimately becomes deleterious and has been linked to Aβ and Tau spreading and propagation, synaptic phagocytosis, and the release of pro-inflammatory cytokines [14,55]. Thus, understanding the engulfment mechanisms used by glial cells is crucial in trying to combat neurodegenerative disorders. Although the cellular steps of the phagocytosis events are well-defined, little is known about the molecular pathways that underlie glial recognition and engulfment targets [56]. The nematode *C. elegans* presents at least two parallel, partially redundant pathways that are involved in the recognition and phagocytosis of apoptotic cells. The first mechanism drives engulfment activity through CED-1, CED-6, and CED-7 pathways, whereas the second one proceeds via CED-2, CED-5, and CED-12 [56]. Taking into account these premises, the effect of EC on the expression of the *ced-7* gene was analyzed. CED-7 is an ABC transporter (ABCA1 or ABCA7 in mammals) that regulates the translocation of various substances, including proteins and phospholipids, across cell membranes in an ATP-dependent manner [56,57]. In addition, as a multispan transmembrane protein, ABCA7 is most abundantly expressed in the microglial cells in the brain [58].

Clinical evidence suggests that excess pro-inflammatory cytokine TNFα is centrally involved in the pathogenesis of AD [59]. In addition to its pro-inflammatory functions, TNFα is a gliotransmitter that regulates synaptic function and mediates the disruption in synaptic memory mechanisms caused by beta-amyloid oligomers [59]. In this study, the expression of the *C. elegans* gene *ZC239.12* was analyzed as an ortholog of human TNFAIP1 (TNF alpha-induced protein 1) [60], whose expression can be induced by TNFα [61].

As for oxidative stress-related genes, two chaperone proteins, i.e., *hsp-16.2* and *hsp-70*, were chosen based on our prior studies in wild-type worms [40,51] and observations from other researchers that these proteins may be modulated by polyphenols. Overexpression of the heat shock protein HSP-16.2 has been described to alleviate proteotoxicity by reducing Aβ_1–42_ oligomers in *C. elegans* AD models [62,63], and several natural bioactive products, such as olive-derived extract [64], paeoniflorin [65], caffeic acid [66], or cranberry extract [67], have shown the ability to induce HSP-16.2 overexpression.

#### 3.5.2. RT-qPCR Analyses

**Figure 5:**
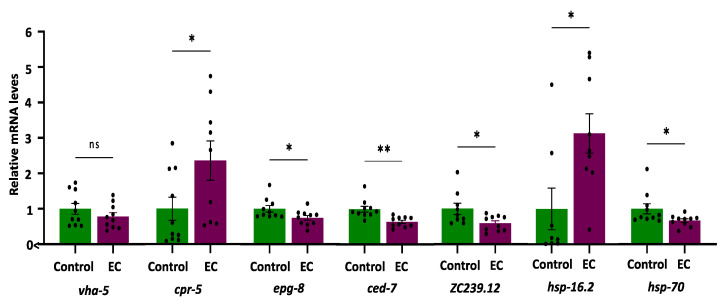
Plots and bars representation of the relative mRNA expression levels of *vha-5*, *cpr-5*, *epg-8*, *ced-7*, *ZC239.12*, *hsp-16.2*, *and hsp-70* genes in CL4176 *C. elegans* cultivated in the absence (controls) and presence of EC (150 μM). Analyses were carried out by RT-qPCR using *act-1* as an internal control. In all cases, ten independent experiments were performed. The results are presented as the mean values ± SEM. Dots represent individual data points. The differences were considered significant at * *p* < 0.05 and ** *p* < 0.01; ns = not significant.

The expression of the selected genes (i.e., *vha-5*, *cpr-5*, *epg-8*, *ced-7*, *ZC239.12*, *hsp-16.2*, and *hsp-70*) was quantified in the CL4176 *C. elegans* strain cultivated in the absence (control) and presence of EC (150 μM). The results are shown in Figure 5.

As for genes related to autophagy, it was found that the expression of vha-5 did not change as a result of treatment with EC, while the mRNA levels of cpr-5 were higher in mutant worms CL4176 treated with EC compared to the control (Figure 5). Cathepsin B was reported to degrade Aβ [68], so enhancing CatB activity could contribute to lower Aβ. On the other hand, a significant descent was observed in the expression of the epg-8 gene in worms cultured in the presence of EC (Figure 5), suggesting that this flavan-3-ol produces a reduction in the accumulation of autophagosomes. Lin et al. [30], using RNAi, demonstrated that the decrease in epg-8 expression in *C. elegans* was a sufficient condition to delay paralysis induced by Aβ since no differences were observed on other genes related to the PI3K complex, such as bec-1 or vps-34. Furthermore, the reduction in epg-8 expression also increased the time interval from the onset, at which 50% of the nematodes were paralyzed compared with the control. Moreover, only reduced expression of epg-8 produced a significant increase in the chemotaxis index in CL2355 worms in relation to controls [30]. All in all, the downregulation of this gene by EC with subsequent reduction in the formation and accumulation of autophagosomes could explain, at least in part, the alleviation of Aβ-induced toxicity and the improvement in the chemotaxis index in worms treated with EC. Studies in *C. elegans* observed that human Aβ_1-42_ expression resulted in autophagosome accumulation in muscle in CL4176 worms [69]. However, as far as we know, there are no studies on the implications of EC in the lysosomal activity, although the effects of another member of the flavan-3-ol family, i.e., epigallocatechin gallate (EGCG), have been explored by other authors. Thus, Dashwood et al. [70] found that EGCG was able to induce lysosomal degradation of cellular components, which could easily have an autophagic nature. Furthermore, Devika and Prince [71] observed it was also capable of stabilizing lysosomal enzymes that are likely to favor degradative processes in the autolysosome. Recently, it was shown that EGCG directly targets intracellular amyloid-β aggregates and promotes their lysosomal degradation [72].

Regarding the ABC transporter *ced-7*, the results show a very significant decrease (*p*-value = 0.001) in the expression of *ced-7* in CL4176 worms treated with EC (Figure 5). The role of ABCA in Alzheimer’s is complex. During AD progression, ABCA7 deficiency can exacerbate neuronal damage and diminish microglial phagocytic capacity, resulting in accelerated Aβ production. By contrast, increased levels of ABCA7 have been described to occur in AD brains and positively correlate with disease severity, i.e., higher expression was associated with more advanced cognitive decline [58,73,74]. According to De Roeck et al. [58], a possible explanation for these opposite observations could be that decreased ABCA7 may contribute to AD pathology in the early stages of the disease; however, it is possible that at later stages, ABCA7 is upregulated as a compensatory mechanism in AD brains. Taking into account the previous discussion, this decrease could be indicative of less cognitive impairment, which would also be consistent with the results obtained in the chemotaxis assays. An increasing number of studies have implicated microglia as the missing link between Aβ accumulation and Tau pathology [14], and an increase in microglial activation has been proposed in AD after Aβ deposition but before the formation of neurofibrillary tangles [75]. In fact, the analysis of transcriptional profiles in Alzheimer’s mouse models has revealed that Aβ pathology activates the expression of many AD genes related to microglia, while this does not happen in Tau-transgenic animals [76]. The downregulation of *ced-7* in worms that express the human Aβ_1-42_ could be essential to avoid the progression of AD. In this context, the inactivation of microglia could be an additional target for flavonoid intervention. Actually, inhibition of microglia activation has been observed in animal models of AD treated with flavonoids [14], and grape seed polyphenols consisting of flavan-3-ols have been shown to reduce Aβ levels, microglia activation, and attenuate inflammation in the brain of an AD mouse model [77]. Similarly, Zeng et al. [21] demonstrated that epicatechin significantly reduced total Aβ, as well as microgliosis and astrocytosis in the brain of Alzheimer’s mice. In light of the obtained results, we hypothesize that the significant descent observed in the expression of *ced-7* (i.e., ABCA1 or ABCA7 in mammals) in CL4176 worms treated with EC, in which a delayed paralysis and an improvement in chemotaxis was produced, could be explained by the use of animals that already had accumulated Aβ after an induction of 32 h, a condition that could be equivalent to a later stage of the disease in humans. In fact, it has been shown that toxic Aβ monomers induce microglial activation and proliferation and that the intervention with flavonoids could reduce the levels of gliosis [14], which would support the results obtained herein with EC.

In the worms treated with EC, a significant descent was produced in the expression of *ZC239.12* (Figure 5), a gene homolog of human TNFAIP1. This observation can be relevant since TNFAIP1 mRNA levels increased robustly in the transgenic CL4176 strain upon temperature upshift, an increase that was also found in post-mortem brain tissue of AD patients [78]. Furthermore, in cultured mouse neuronal cells, TNFAIP1 was induced by and promotes Aβ_25–35_ induced apoptosis and inhibits Akt/CREB signaling, thereby contributing to neurotoxicity [79]. Similar to our findings, Xin et al. [31] found that the treatment of the alkaloids galanthamine, hemanthidine, and 1,2-di-*O*-acetyllycorine significantly reduced the expression of *ZC239.12*, concluding that these compounds could limit inflammation and stress-related cellular damage caused by Aβ toxicity in transgenic CL4176 worms. All in all, the observed decrease in TNFAIP1 expression induced by EC can be important to explain its apparent neuroprotective effects, as TNFAIP1 could be a crucial pathological biomarker of AD and may play an important role in regulating AD progression, being a promising therapeutic target for AD, as also pointed out by Xiao et al. [79].

Finally, to check whether the heat sock stress response could be one of the mechanisms underlying the protective effect of EC against Aβ proteotoxicity, the expression of *hsp-16.2* and *hsp-70* genes was analyzed. The results obtained showed an increased overexpression of *hsp-16.2* in CL4176 worms, while *hsp-70* expression was reduced by 21% with respect to control worms (Figure 5). The existence of a different behavior in the expression of these two genes in response to different bioactives was noted in other studies. Thus, paeoniflorin was seen to upregulate *hsp-16.2*, whilst it did not change the expression of *hsp-70* in the CL4176 and wild-type N2 strains [65]. Similarly, caffeic acid was only able to upregulate *hsp-12.6* among six studied heat shock proteins (*hsp-3*, *hsp-16.1*, *hsp-16.41*, *hsp-17*, and *hsp-70*) in N2 worms [80]. A previous work of our group demonstrated that the treatment of the N2 strain with EC increased expression of *hsp-16.2* after thermal stress, an effect that was more accentuated in older worms [40]. This observation could be relevant because heat shock protein levels are known to decrease in aged animals, leading to an increase in unfolded proteins [81]. Fonte et al. [62] showed that *shsps* (i.e., small hsps) can bind intermediate multimers, reducing the formation of toxic oligomer species and playing a role in a range of neurodegenerative diseases associated with toxic protein aggregates. A decrease in small heat shock protein function with age could also contribute to the age-dependent onset of these diseases [62]. Thus, the increase in the expression of *hsp-16.2* observed both in old worms [40] and in AD transgenic worms, in which HSP are already over-expressed, could contribute to explaining the neuroprotective effects of EC. What is more, chaperone activation could have a greater influence on the ability of EC to maintain protein-folding homeostasis in worm models of AD than other mechanisms, such as proteasomal activity.

## 4. Conclusions

The assays carried out in *C. elegans* models of Alzheimer’s disease provided insights into the beneficial effects of epicatechin against Aβ-induced toxicity. EC (150 µM) significantly decreased the rate of worm paralysis induced by Aβ accumulation in the CL4176 strain and extended lifespan and increased survival in the CL2006 strain, both expressing human Aβ_1-42_ and exhibiting paralysis with age. EC also improved the chemotaxis behavior in CL2355 worms expressing Aβ_1-42_ in neurons, suggesting neuroprotection against amyloid toxicity. In order to obtain clues regarding the potential molecular mechanisms underlying these effects, the expression of some genes related to signaling pathways involved in endogenous cell defences was analyzed by RT-qPCR. It was found that treatment with EC induced higher expression of *cpr-5*, an ortholog of human cathepsin B and an endopeptidase that plays a role in Aβ degradation, so that enhanced *cpr-5* activity could lead to decreased Aβ levels. By contrast, EC downregulated *epg-8*, a gene related to autophagosome formation. A reduction in autophagosome accumulation may contribute to the alleviation of Aβ-induced toxicity. EC also reduced the expression of ZC239.12, a *C. elegans* homolog of human TNFA1P1 associated with inflammation and apoptosis, while it led to upregulation of *hsp-16.2*, a small heat shock protein (sHSP) implicated in proteotoxicity alleviation. Furthermore, EC treatment resulted in a significant decrease in the expression of *ced-7* (ABCA1 or ABCA7 in mammals). Increased expression of this gene was positively correlated with disease severity in later stages of human AD so its downregulation could contribute to explaining the reduced damage observed in EC-treated worms. We are aware that gene expression data are not enough to fully explain the observed effects. After transcription, the mRNAs must be translated to proteins. Nevertheless, we believe that the observations made, although preliminary, are important, as they demonstrate the ability of EC to act at a molecular level. Certainly, further studies must now be addressed to check the amount and activity of proteins produced. All in all, the outcomes of this study demonstrate that epicatechin possesses potential neuroprotective properties, which might be related to its ability to modulate the expression of genes related to multiple intracellular pathways, including autophagy, microgliosis, inflammation, stress damage, and heat shock signaling. Further research is, however, required to continue delving into the underlying mechanisms of the protective effects of EC and their potential clinical relevance in the context of Alzheimer’s disease treatment.

## Figures and Tables

**Table 1:** Delay in the time of observation of paralysis in CL4176 worms grown in the presence of different EC concentrations.

Treatment	Average Time of Non-Paralyzed Worms ^1^ (Hours ± Standard Deviation)	*p*-Value ^2^ (EC vs. Control)
Control	30.6 ± 0.17	
EC 200 µM	30.9 ± 0.27	0.574
EC 150 µM	31.3 ± 0.15	<0.001 ***
EC 100 µM	30.7 ± 0.15	0.421
EC 50 µM	30.6 ± 0.11	0.686

^1^ Time at which 50% of the worms are not yet paralyzed. ^2^ Statistical significance of the difference between EC-treated and untreated worms (control) was calculated by the log-rank test analysis. *** Statistically significant at *p* < 0.001.

**Table 2:** Life duration of *C. elegans* CL2006 strain grown at 20 °C in the absence and presence of EC (150 µM) in the culture medium.

Assay	Mean (Days) ^1^	*p* vs. Control (Log-Rank)	Maximum 10% (Days) ^2^	*p* vs. Control (Anova)
Control	18.2 ± 0.52		30.2 ± 1.21	
EC 150 µM	20.6 ± 0.54	0.000	32.6 ± 1.71	0.000

^1^ Results are expressed as mean ± standard deviation (n = 100 worms/experiment). The results were obtained from three independent experiments. ^2^ Maximum lifespan was determined as the mean lifespan of the longest-living 10% of each population. Statistical significance was calculated by log-rank testing for mean life and using one-way analysis of variance for maximum lifespan. In both cases, the differences were considered significant at *p* < 0.05.

## Data Availability

The data presented in this study are available on request from the corresponding author.

## Supplementary Materials

The following supporting information can be downloaded at https://www.mdpi.com/article/10.3390/antiox13010079/s1, Table S1: Oligonucleotide primers used in qRT-PCR studies.
