# Supplementary material for: Insights into the Neuroprotective Potential of Epicatechin: Effects against Aβ-Induced Toxicity in Caenorhabditis elegans
Source: Antioxidants (Basel). 2024 Jan 8;13(1):79. doi: 10.3390/antiox13010079 (PMC10812808; doi:10.3390/antiox13010079)
Supplement: Supplementary file 1 [file antioxidants-13-00079-s001.zip › antioxidants-2737732-supplementary.pdf]

Table S1: Oligonucleotide primers used in qRT-PCR studies.

Gene	Forward (5′-3′)	Reverse (5′-3′)	Accession number	References
act-1	CCAGGAATTGCTGATCGTATG	GGAGAGGGAAGCGAGGATAG	NM_073418.9	[29]
vha-5	CTTCATGGAAACGCGACTGT	CGGTAACGAACACCATGTGC	NM_068998.10	[30]
cpr-5	CTCCGACGCTATTCCAGACC	GCGTAGGCGGTAGATCCAAA	NM_070982.7	[30]
epg-8	GCGGTAAACGCTACACAAAGA	CCATCCGCTGAGATTCCTGG	NM_170914.6	[30]
ced-7	GAAGTGGCAGATTCCGAAAGA	TCAAGGGAGGACTGAGCTAATA	NM_001026054.4	Designed by the research team
ZC239.12	CCAGAAGAATCCCCATACGA	TCCTCCTCCAACTTTTCCAAA	NM_062072.3	[31]
hsp-16.2	CTGCAGAATCTCTCCATCTGAGTC	AGATTCGAAGCAACTGCACC	NM_071106.8	[32]
hsp-70	CAAGACTTTGGAGCCGGTTG	GGAGCAGTTGAGGTCCTTCCC	NM_060084.6	[30]
